# Use of scientific social networking to improve the research strategies of PubMed readers

**DOI:** 10.1186/s13104-016-1920-y

**Published:** 2016-02-18

**Authors:** Pavel Evdokimov, Alexey Kudryavtsev, Ekaterina Ilgisonis, Elena Ponomarenko, Andrey Lisitsa

**Affiliations:** Knowledge Technologies Ltd, Moscow, Russia; qB Ltd, Moscow, Russia; Institute of Biomedical Chemistry, Moscow, Russia

**Keywords:** Bioinformatics, Crowdsourcing, Social network, Text-mining

## Abstract

**Background:**

Keeping up with journal articles on a daily basis is an important activity of scientists engaged in biomedical research. Usually, journal articles and papers in the field of biomedicine are accessed through the Medline/PubMed electronic library. In the process of navigating PubMed, researchers unknowingly generate user-specific reading profiles that can be shared within a social networking environment. This paper examines the structure of the social networking environment generated by PubMed users.

**Methods:**

A web browser plugin was developed to map [in Medical Subject Headings (MeSH) terms] the reading patterns of individual PubMed users.

**Results:**

We developed a scientific social network based on the personal research profiles of readers of biomedical articles. A browser plugin is used to record the digital object identifier or PubMed ID of web pages. Recorded items are posted on the activity feed and automatically mapped to PubMed abstract. Within the activity feed a user can trace back previously browsed articles and insert comments. By calculating the frequency with which specific MeSH occur, the research interests of PubMed users can be visually represented with a tag cloud. Finally, research profiles can be searched for matches between network users.

**Conclusions:**

A social networking environment was created using MeSH terms to map articles accessed through the Medline/PubMed online library system. In-network social communication is supported by the recommendation of articles and by matching users with similar scientific interests. The system is available at http://bioknol.org/en/.

**Electronic supplementary material:**

The online version of this article (doi:10.1186/s13104-016-1920-y) contains supplementary material, which is available to authorized users.

## Background

Crowdsourcing offers new opportunities for examining the life sciences [1, 2]. There are a number of professional networks, e.g., LinkedIn or ResearchGate, based on a compendium of published articles that can be used to construct user profiles. Registered users can see their collaborations organized into graphs according to number of co-published papers. In the projects Nature Network and PubMed Commons, participants are invited to comment on articles. Such a mechanism is supported in several other reference management systems such as Mendeley [3].

In recent years several propositions for fostering biomedical research through the use of social networking have gained strength [1]. First, and most importantly, users can share published articles with fellow researchers. In the biomedical field, however, most scientific articles represent a collective achievement and the list of co-authors on a paper lacks specific information about individual contributions. Second, scientific crowdsourcing necessarily involves posting interesting papers at the aggregate portal. This activity adds momentum to the research effort because the user must decide ultimately whether a paper is worth reading. Third, there exist microenvironments of scientific topics supported by the reference manager, as in the case of Mendeley.

We set up various functional categories of current social scientific services. LinkedIn and other similar services typically put together people who want to “keep in touch”, within a certain professional venue. These functional scientific social networks draw attention to past scientific achievements of users and often serve as recruiting vehicles for interaction between principal investigators (PIs). Posting a paper requires some intrinsic decision-making regarding whether or not the paper is interesting; this analytical approach requires examining the present “state of the art” (CiteULike). It can be hypothesized that reference-based microenvironments are biased because of the necessary focus of the task, e.g., preparing the reference list for the paper, dissertation or grant application. The limited nature of the overall goal, i.e., posting the paper where it can be available to other researchers, creates a mixture of factors, past, present and future (Mendeley), with individual contributions becoming computationally indistinguishable.

The concept of creating the BioKnol software is based on non-equivalence of social contributions of the scientists and scientific researchers as those who generate new knowledge: i.e., those who consume scientific information.

1 $${\text{S }} \ll {\text{ SR}}$$

where S stands for the number of articles from the people actually interested in pure science, while SR denotes those contributions whose authors do science as a job. From Eq. (1) it was supposed that existing social networks are biased towards scientific researches (SR): these are using science to build their careers.

Alternatively to strengthening disequilibrium in the Eq. (1) we proposed a scientific social network where a user is associated with a profile of articles browsed on the Internet. Such type of browsing is relevant to scientific curiosity rather than to building a career in science. By limiting the system to a biomedical knowledge domain, we developed the mechanism of matching the frequencies of occurrence of Medical Subject Headings (MeSH) to create social groups of researchers working in a similar field. Also, the mechanism of sharing the most important scientific articles within groups of users was implemented to the system.

## Methods

The scientific social network was implemented using a web browser plugin and the Dot NET server. The plugin could be installed by the users of Mozilla Firefox, Google Chrome, Internet Explorer and Safari. After installation, the plugin operates in anonymous mode. By clicking the plugin the user could opt for being authorized or reject further usage of the plugin. The plugin was connected to the server through the protocol (see figure in Additional file 1): (1) transmit the current URL to the server; (2) receive if URL matched to the list of the scientific websites appointed by server administrator; (3) display if the website is monitored or not and provide an option for a user to include current site into the list for monitoring; (4) notify the user if the current web page could be unequivocally translated into the article identifier, either digital object identifier (DOI) or PubMedID. The DOI was resolved to PubMed ID by requesting an article title and two first authors via CrossRef.

URL and HTML analysis is based on consequent application of parsing rules. As a result, two decisions are made: (1) whether the web page is an article, (2) whether there is DOI and/or PubMedID on the web page.

Parsing rules are defined by the knowledge managers in terms of regular expressions. There are “common” parsing rules enabling recognition of DOI/PubMedID at any web page and “website-specific” customized parsing. If page expressions (Table 1) are recognized as an article, website-specific parsing rules apply. If they fail, common rules are probed. If they also fail, HTML is saved for later manual inspection. The knowledge manager defines parsing rules as far as visits to web pages; newly developed parsing rules are applied against previously collected files.

**Table 1:** Regular expressions used in BioKnol

URL	Expression	ID type
Any web page	(?<=http://dx.doi.org/).{1,70}(?=</p >)	DOI
Pubmed.org	(?<=ncbi.nlm.nih.gov\/pubmed\/)\d{1,15})	PubMed ID
Nature.com	(?<=meta name=\”citation_doi\” content=\”doi:)(.|\\s)*?(?=\”>)	DOI

An approach of regular expressions is supported by the constant necessity to monitor rate of article IDs recognition for specific websites. Knowledge manager adjusts parsing rules for specific websites if ID-recognition rate drops (this could be caused by changes in previously HTML layout).

## Results

BioKnol monitors 384 websites where 144 are with customized PubMed-regular expressions and 176 are with customized DOI-regular expressions. For 5 % of the most frequently accessed sites including pubmed.org, Elsevier.com, Springer.com, and others, regular expressions were manually developed. Over 1500 registered users joined the PubMed-based social network during the period of 2011–2013 (mostly from Russia, the promo-site was provided in the Russian language). Out of that there were 9.8 % registered users, while the others remained anonymous. We evaluated the users’ activity as a ratio of the number of days since installation of the plugin to the number of days, when at least one access was detected. As for the newcomers, this index was rather high—over 50 %, the value for those who used the system for more than a year was below 3 %. We also noticed that there are about 2 % of diligent readers who read 1.5 articles per day on average, and also many passive readers who viewed at least 1 article in 10 days. In addition to PubMed about 98 % of the users accessed the articles from the dedicated sites of the top 100 of resources of scientific literature. These were well known content aggregators, like ScienceDirect, or the sites of the leading journals in the field: Nature, Cell, Oncogene, PNAS etc.

**Fig. 1:**
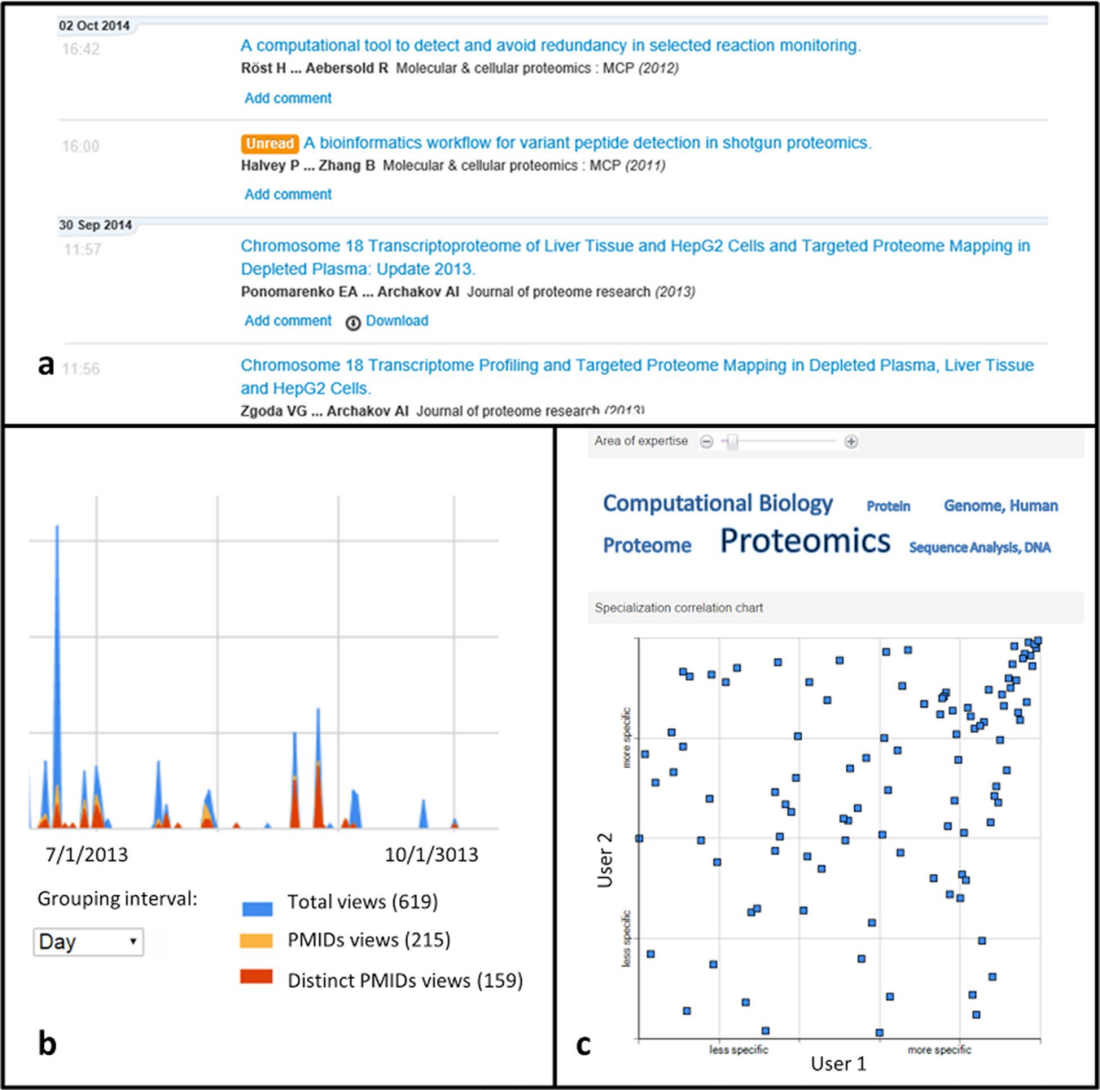
The scientific social network: **a** activity feeds recording accessed articles; **b** time-scale of user reading activity; and **c** matching reading profiles of two users

The scientific social network was developed to share the reading profiles of the users. At the front end of the network the user can get the activity feed as shown in Fig. 1a. The feed lists the articles recognized by the system’s server by their DOI or PubMed identifiers. Each item in the list corresponds to single access to an article, indicating the first and last authors, the title and the year of publishing. The activity feed includes the articles viewed by a user and also the articles viewed by the network users s/he has subscribed for. Article viewing is supported by subscription mechanism, as described further below.

Activity feed provides information concerning how many times the item (article) was revisited and if there were any comments on a particular article. The citation could be recommended to any network user and total number of recommendations is displayed in the feed.

It is possible to switch from the activity feed to viewing a certain article at an article view page. The article view expands the comments and shows the users who also have browsed the same article. Selecting a person from the list of the article’s co-readers provides access to the activity feeds of the colleagues. The navigation through the activity feed of a colleague enables one to find out other relevant articles which were skipped in the reading background.

Browsing the reading profiles, a user can subscribe to a particular feed of another member of the BioKnol network. Subscriptions enrich activity feed with the items viewed by other users. It could be used in both ways: first, when the leader is enriching the knowledge domain by viewing the reading of the group members. Otherwise, the research group could follow the information of a member who is selecting related articles.

In the developed scientific social network a user is offered an activity graph where the accesses to the citations are plotted as a function of time. As all of the accessed citations are automatically mapped to PubMed identifiers, a user has an option to distinguish the fraction of newly accessed articles to the fraction of the previously accessed ones over time (Fig. 1b).

While working with the system it is possible to observe the MeSH profile of each user. Articles in the user’s activity feed are queried to PubMed to retrieve the list of MeSH associated with the abstracts. The occurrence frequencies of MeSH in the users’ dataset of articles are visualized as a tag cloud (Fig. 1c).

In order to facilitate the scientific matching the developed social network enables comparison between the user’s profiles. As shown in Fig. 1c the results of the comparison are presented as a scatter plot where axes correspond to the frequency of occurrence of the MeSH descriptors of user 1 and user 2. Dots are assigned to the specific MeSH; descriptors are displayed when mouse steps over the dot. In the upper right part of the plot there are descriptors occurring with high frequency for both users. The high density of the dots in the upper-right part indicates that generally two users share similar profiles. The upper-left (as well as bottom-right quarters) of the plot indicates the specific differences between the reading profiles.

Implemented social network provides three tools for supporting communications and expanding the community:

1. Who else has accessed the same article which I am interested in?
2. Who has a MeSH profile relevant to my activities?

Each of the listed tools fulfills one and the same mechanism to match the users by their interests, to allow them to read the activity feed of the matching partner and finally to make a subscription for the interesting feeds.

**Fig. 2:**
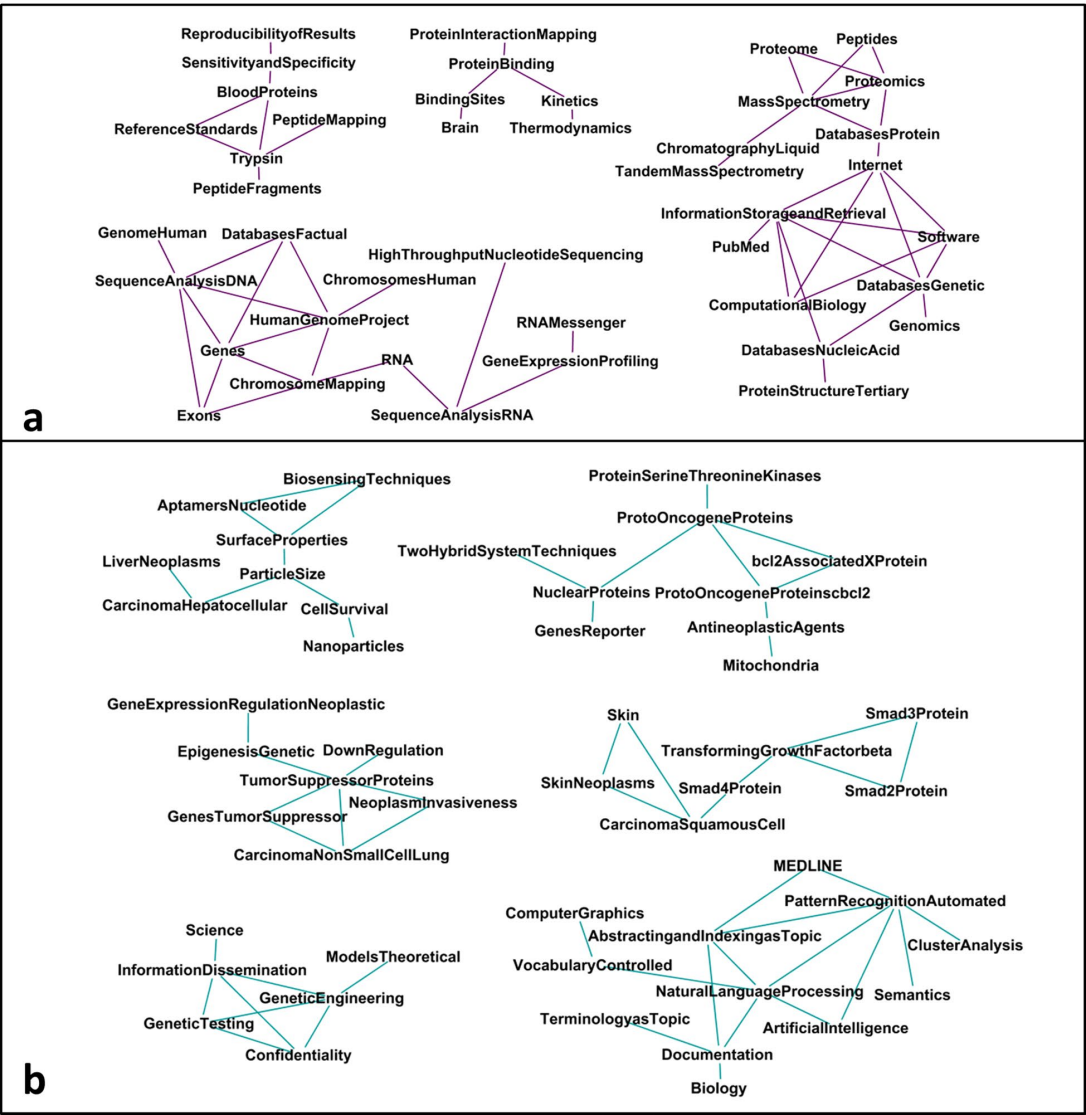
The semantic networks of current (**a**) and future (**b**) fields of the research of a BioKnol user: **a** MeSH descriptors for intersection of BioKnol and Mendeley profiles; **b** MeSH descriptors unique for BioKnol

We have compared BioKnol and Mendeley profiles after 3-years of simultaneous usage. The semantic networks were constructed according to the previously described method based on the frequency of co-occurrence of MeSH descriptors [4]. The difference between BioKnol and Mendeley profiles is shown in Fig. 2, where Fig. 2a shows MeSH descriptors common for BioKnol and Mendeley profiles. Semantic networks at Fig. 2 consist of clusters which correspond to the method-specific MeSH descriptors, i.e., proteomic and NGS. It could be proposed that Mendeley creates a corpus of papers which are relevant to the scientific duties of the user, as in Life Sciences the researcher is a person that has to produce the result by way of the published paper. Main function of Mendeley is to cite papers from the library therefore Mendeley profile represents papers selected for citing in grant reports and specific publications written for hire, in concordance with the above assumption the large cluster at Fig. 2 refers to proteomic profiling using mass-spectrometry and database searching because it is based on publications selected for citing in annual report of Chromosome-centric Human Proteome Project [5]. Thus, Mendeley profile represents professional competence of the scientist in a particular rather narrow field of research.

We observed that BioKnol delivers a different content from Mendeley. The semantic network based on MeSH descriptors, unique for BioKnol profile (i.e., absent in the Mendeley profile) is presented at Fig. 2b. The BioKnol network consists of six clusters indicating other methods, not visible from Medeley; these are natural language processing and bioethics regulations in genetic screening. The rest of the clusters are evidently linked to neoplasms including lung and liver carcinomas and skin tumors. Besides, the association of particular genes (Bcl2 and SMAD family) to the reading is captured from BioKnol (Fig. 2b) but not from the Mendeley network (Fig. 2a). Speculatively, network constructed using BioKnol data represents additional interests, which could be the core of the future research strategies.

## Conclusions

In our model of the scientific social network access to articles is broadcast to all PubMed users who have previously accessed the same item. We created an environment that has a crowdsourcing effect due to the fact that quite a large number of researchers are regularly accessing scientific articles [1]. The impact of those who browse articles is underestimated. The current paradigm relies on a relatively small proportion of individuals who are eager to write articles [6, 7], while other scientifically educated people remain outside of mechanisms of professional socialization.

Possible frustration in sharing the reading activity could occur if this mechanism of social networking is used to steal new scientific ideas. However, it is difficult to grasp an idea just by looking into the sequence of accesses to articles. At the same time, the proposed social network could provide support to some ideas with limited viability. If two people independently have reproduced the same pattern of reading, it might be an indication of reliability of the idea that stands behind the reading pattern. In such cases, both users could be notified of this statistically reliable fact; it is then up to their discretion whether they thereafter choose to compete or collaborate [8].

## Availability and requirements

- *Project name* Scientific social network
- *Project home page*http://bioknol.org/en/
- *Operating system(s)* Windows Server 2008 or higher
- *Programming language* .NET
- *Other requirements* SQL Server 2008 Standard Edition or higher; Dot NET Framework 4.0 or higher
- *License* The source code of the server application is distributed under the Creative Commons and is available at: https://bitbucket.org/evdokp/bioknol_pub/src/
- *Any restrictions to use by non*-*academics* none

## Additional file

10.1186/s13104-016-1920-y BioKnol—Systems Architecture (Figure).

## Abbreviations

DOI: digital object identifier
MeSH: Medical Subject Headings
