# Supplementary material for: Use of scientific social networking to improve the research strategies of PubMed readers
Source: BMC Res Notes. 2016 Feb 18;9:113. doi: 10.1186/s13104-016-1920-y (PMC4758102; doi:10.1186/s13104-016-1920-y)
Supplement: Supplementary file 1 — 10.1186/s13104-016-1920-y BioKnol—Systems Architecture (Figure). [file 13104_2016_1920_MOESM1_ESM.pptx]

## Slide 1
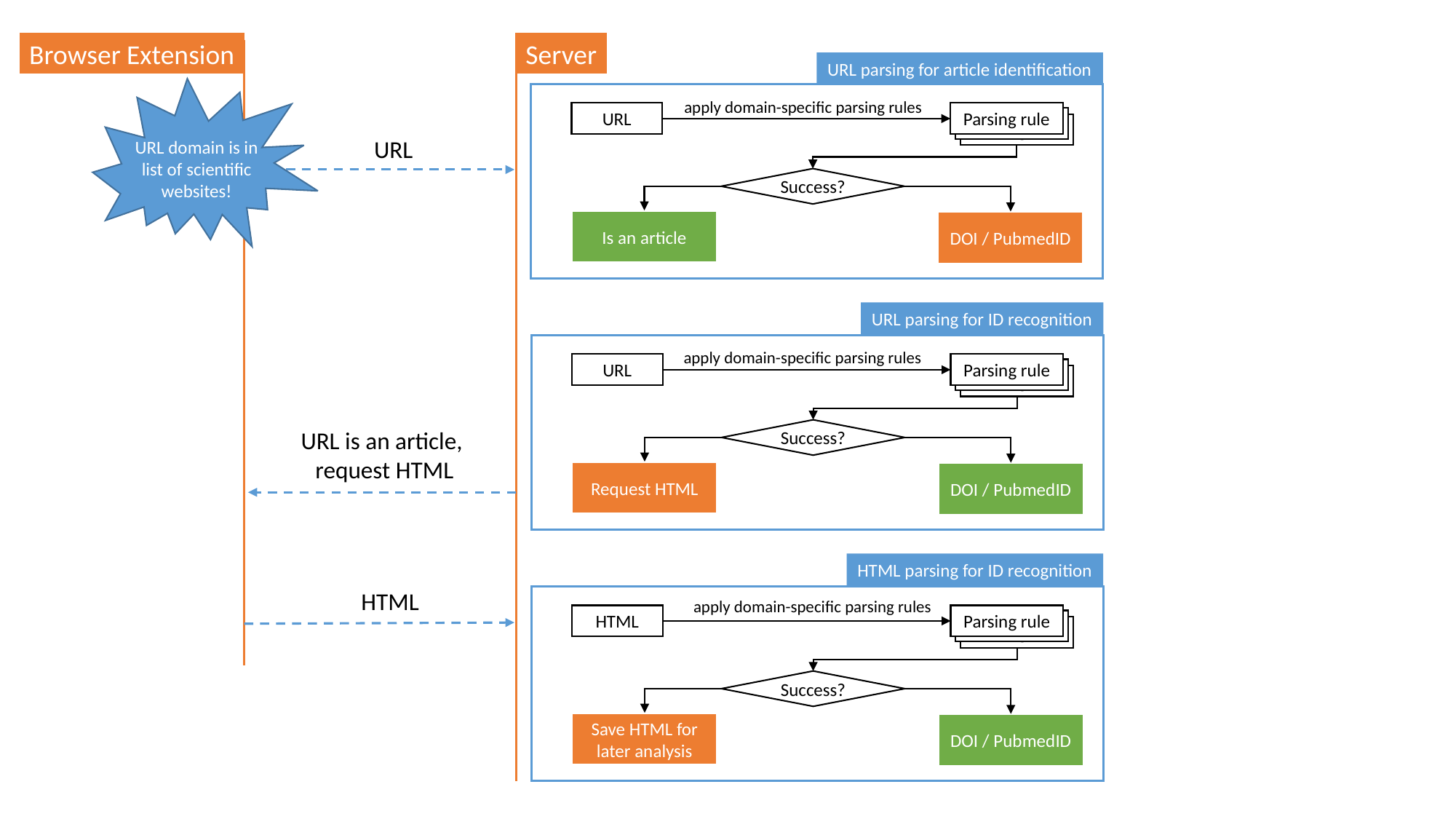

Browser Extension
Server
URL parsing for article identification
apply domain-specific parsing rules
URL
Parsing rule
Parsing rule
Parsing rule
Success?
Is an article
DOI / PubmedID
URL domain is in list of scientific websites!
URL
URL parsing for ID recognition
apply domain-specific parsing rules
URL
Parsing rule
Parsing rule
Parsing rule
Success?
Request HTML
DOI / PubmedID
URL is an article,
request HTML
HTML parsing for ID recognition
apply domain-specific parsing rules
HTML
Parsing rule
Parsing rule
Parsing rule
Success?
Save HTML for later analysis
DOI / PubmedID
HTML
